# Circulating Tetraspanins: From Markers to Mechanisms Driving Systemic Exercise Adaptation

**DOI:** 10.1093/function/zqad048

**Published:** 2023-09-01

**Authors:** Darby S Easterday, Daniel S Lark

**Affiliations:** Department of Health and Exercise Science, Colorado State University, Fort Collins, CO 80523, USA; Department of Health and Exercise Science, Colorado State University, Fort Collins, CO 80523, USA

**Keywords:** exosome, extracellular vesicle, obesity, metabolic disease, skeletal muscle, adipose tissue, endocrinology, tetraspanin

Exercise improves cardiometabolic health through a range of systemic [ie, beyond working skeletal muscle (SkM)] mechanisms typically attributed to small molecules and peptide hormones. Recent discoveries have shown that the abundance and cargo of circulating small extracellular vesicles (sEVs) like exosomes are altered by exercise, but linking these changes to SkM-derived systemic exercise adaptations has been challenging. A key barrier to linking SkM sEVs to exercise adaptations is determining which of the hundreds of molecules that may be transported by SkM sEVs have functional relevance in the context of exercise. One surprisingly untested strategy is to start with the most abundant sEV cargo. Tetraspanins like CD81 are transmembrane protein hallmarks of sEVs. To date, CD81 has only been described as an sEV marker, not an instrument of sEV function. However, ∼30 yr of research has established CD81 as a transmembrane adaptor protein that influences a variety of cellular functions by altering the organization of receptor proteins within membranes. Multiple groups have recently shown that exercise increases the abundance of circulating sEVs containing CD81^1,2^ (CD81^+^ sEVs), which suggests that exercise may increase the delivery of CD81 to recipient cells. Another recent discovery has shown that CD81 supports the preservation of adipose tissue metabolic health during obesity,^3^ but it is not known to what extent CD81^+^ sEVs contribute to this phenomenon. Moreover, no studies to date have described the impact of obesity on the abundance of circulating CD81^+^ sEVs or what effects circulating CD81 protein may have on recipient cells. In this Opinion, we combine contemporary evidence on exercise-induced circulating sEVs and the metabolic effects of CD81 in adipocytes with historical evidence on cellular CD81 function to propose SkM-to-adipose tissue trafficking of CD81 in sEVs as a novel mechanism driving exercise adaptations.

**Figure 1.**
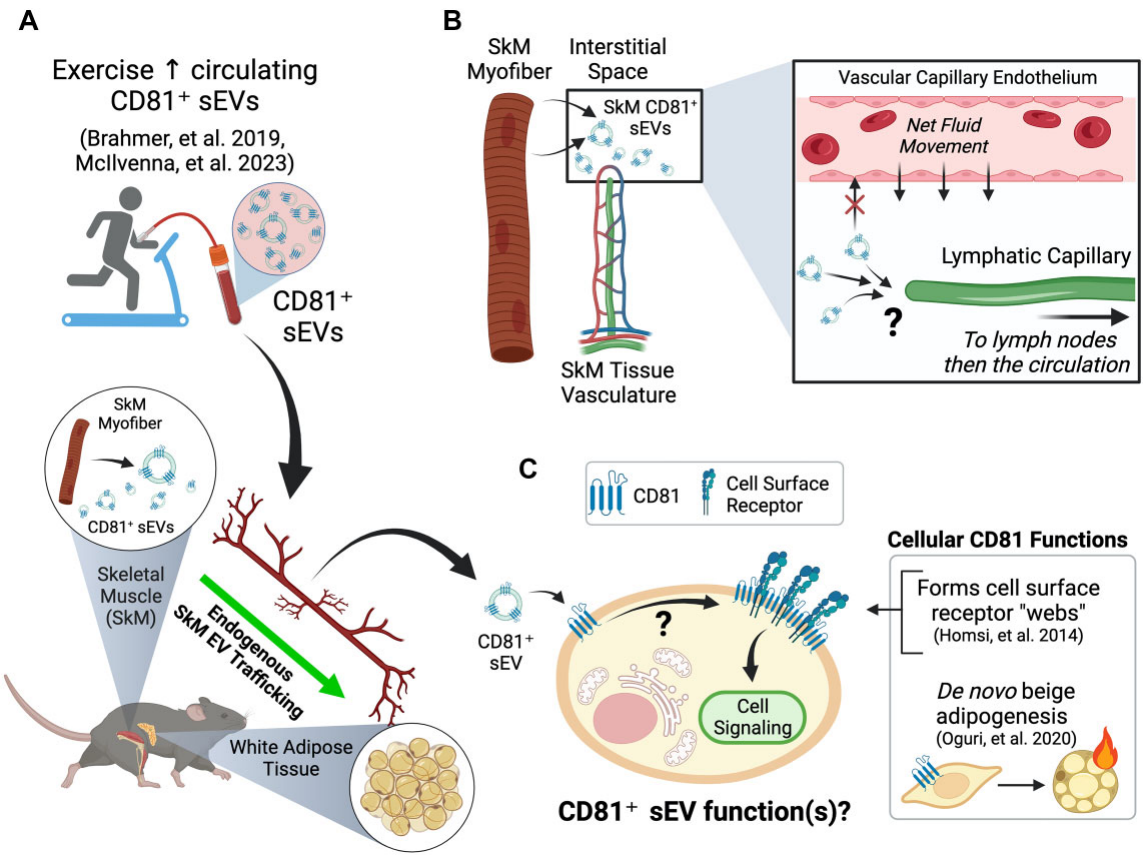
Evidence linking the cellular functions of CD81 to sEVs during exercise. (A) A single bout of continuous aerobic or high-intensity interval training increases CD81^+^ sEVs in the circulation in human subjects. In mice, SkM myofibers are a major source of CD81^+^ sEVs reaching the circulation. Endogenous SkM sEVs accumulate in adipose tissue. (B) sEVs are too large to cross endothelial tight junctions, and Starling forces across the capillary endothelium push plasma into the interstitial space. SkM EVs may be taken up by lymphatic capillaries that collect interstitial fluid (which contains sEVs) and transported back to the blood. (C) In cells, CD81 modulates signaling pathways by forming “webs” consisting of CD81 and a variety of cell surface receptors, including integrins (shown). CD81 is necessary and sufficient for de novo beige adipogenesis, which has health-promoting effects on energy expenditure. Future studies could examine whether CD81^+^ sEVs are capable of regulating signaling pathways and physiological functions already attributed to cell-intrinsic CD81. Figure created with Biorender.com.

*What evidence links CD81* ^+^ *EVs to the physiology of exercise?* The study of sEVs and their cargo during exercise is a popular but convoluted area of investigation. Inconsistent isolation methods, various exercise modalities, and different model systems have hindered progress and precluded consensus in the field. This convolution is worsened by a lack of tools to study cell-type-specific (eg, SkM) sEV populations in vivo. Despite these challenges, some of the strongest evidence linking sEVs to exercise is through CD81. Both progressive^1^ and high-intensity interval training^2^ exercises acutely increase the circulating abundance of CD81^+^ sEVs of human subjects (Figure 1A). Brahmer et al.^1^ measured CD81 protein from isolated plasma sEVs from young, healthy, and active men at three time points of an incremental cycling exercise protocol: baseline (immediately prior to exercise), during exercise once respiratory quotient (RQ) exceeded 0.9 (which the authors equated to a “moderate” exercise intensity), and immediately after (<2 min) cessation of exercise at maximal workload. The authors found that both moderate (RQ ∼ 0.9) and maximal intensity exercises increase CD81 protein abundance in circulating plasma sEVs. A more recent study from MclIvenna et al.^2^ quantified individual circulating plasma CD81^+^ sEVs during exercise. In this study young, active, healthy males and females performed high intensity interval training (HIIT) exercise (4 × 30 s of cycling at 200% of each participant’s predetermined maximal power output). Plasma was collected immediately prior to exercise and <30 s after the final exercise interval. CD81^+^ sEVs were captured on a microfluidics chip using a CD81-specific antibody and directly quantified using fluorescence detection. Using this more rigorous and direct approach to quantify protein-specific sEV populations, the authors found that HIIT increases the number of circulating CD81^+^ sEVs. To build upon these promising findings, future studies could explore how long CD81^+^ sEVs are present in the circulation after exercise, identify the cells (ie, SkM myofibers) that secrete CD81^+^ sEVs during exercise, and discover what function(s) they have on recipient cells.

It is intuitive and convenient to attribute the effects of exercise on circulating sEVs to SkM myofibers, but there is limited direct evidence to support (or refute) this idea. This knowledge gap exists because there is no known sEV cargo that is exclusive to SkM myofibers, and therefore establishing the mere existence of SkM sEVs in the circulation has been challenging. To address this knowledge gap, our group used a fundamentally different strategy. Instead of trying to find an endogenous SkM-specific sEV cargo, we used Cre-dependent expression of a fluorescent reporter protein in mice to track SkM myofiber-derived sEVs.^4^ With this approach, we found that SkM secretes CD81^+^ sEVs, which reach the circulation. It is not yet known if, and to what extent, exercise increases circulating SkM CD81^+^ sEVs or where they might accumulate. However, recent work by Ismaeel et al. found that sEVs containing the muscle (SkM and cardiac)-specific miRNA, miR-1, accumulate in white adipose tissue.^5^ These data show that exercise increases circulating CD81^+^ sEVs, some of which may originate from SkM myofibers, and suggest that white adipose tissue may be a target organ of SkM sEVs secreted during exercise.

*How might SkM EVs reach adipose tissue?* It is not yet clear how sEVs travel from tissues to the blood, but the known anatomical features of the body offer important clues. That is, sEVs are much larger (50-200 nm diameter) than tight junctions of the SkM capillary endothelium (2-3 nm diameter), which should not allow for the passive transendothelial flux of sEVs from the SkM interstitium directly into the circulation (Figure 1B). Moreover, Starling forces dictate that an intact cardiovascular system forces fluid from the capillary endothelium into the interstitial space and subsequently into the lymphatic circulation. Therefore, we believe it is most likely that SkM sEVs reach the blood through the lymphatic circulation. There is experimental evidence for both lymphatic and transendothelial flux of exogenous sEVs, but no studies to date have examined SkM sEV trafficking or established a mechanism of endogenous sEV trafficking from tissues to the blood. Establishing how sEVs from SkM myofibers (and other tissue resident cell types) reach the blood may improve the design and development of tissue-derived sEVs as disease biomarkers and therapeutics.

The mechanisms regulating sEV uptake into cells are also incompletely defined. Previous work has shown that adipocytes uptake endothelial cell-derived sEVs in vitro and in vivo and this process is impaired by overnutrition.^6^ In vitro studies have shown that CD81 protein delivered to cultured cells via sEVs is functional,^7,8^ which suggests that intercellular transfer of CD81^+^ sEVs in vivo allows recipient cells to “adopt” functions attributable to CD81. Future studies are needed to firmly establish the physiological function(s) of CD81^+^ sEVs on metabolic health and disease outcomes.

*What are the cellular functions of CD81?* CD81 is a transmembrane protein that indirectly enhances adhesion receptor-mediated cellular processes via molecular “webs” [or tetraspanin-enriched microdomains (TEMs)] that aggregate receptors at the cell surface^9^ (Figure 1C). The most thoroughly described mechanism of CD81 action is its ability to lower the threshold to activation of B cells and T cells through physical interactions with CD19/CD21 and CD4/CD8, respectively. CD81 is also known to interact with integrin receptors that not only regulate cellular behavior, but are also involved in metabolic disease etiology. Recent discoveries by Oguri et al. have demonstrated that CD81 preserves cardiometabolic health via integrin-dependent signaling in white adipose tissue.^3^ In their study, the authors demonstrated that CD81 is both necessary and sufficient for de novo beige adipogenesis,^3^ an adaptation that increases energy expenditure and may be a treatment for obesity and other metabolic diseases. In this same study, the authors discovered that whole-body deletion of CD81 in mice worsened the consequences of diet-induced obesity in white adipose tissue (eg, fibrosis, inflammation, and insulin resistance) by disrupting integrin signaling. Finally, the authors demonstrated that a low abundance of CD81^+^ adipocyte progenitor cells within resident white adipose tissue depots is associated with impaired glucose homeostasis, adiposity, and hypertension in human subjects. The authors did not examine a role for CD81^+^ sEVs in this study, and it is not yet known how metabolic disease influences the circulating abundance and trafficking of CD81^+^ sEVs. However, the acute exercise-induced increase in circulating CD81^+^ sEVs may cause greater delivery of CD81 protein to adipose tissue (and other tissues), thereby contributing to exercise adaptations. Future studies could seek to define the ability of CD81^+^ sEVs to positively modulate cell signaling pathways necessary for cardiometabolic health.

As the body of literature on sEVs continues to grow, it is important to recognize that the systemic effects of sEVs and their cargo are the product of trafficking, abundance, and intracellular function(s). Because CD81 is such an abundant sEV protein, it is possible that its systemic physiological impact when trafficked in sEVs could meet, or exceed, the effects of miRNAs and other scarce sEV cargo that have more tractable molecular mechanisms. The ability of CD81 to enhance the sensitivity of cell signaling pathways makes it a particularly interesting target for cardiometabolic diseases that are characterized by resistance to second messenger signaling. Based on the evidence provided here, we hypothesize that sEVs contribute to systemic adaptations to exercise via SkM-to-adipose trafficking of CD81. This hypothesis could be tested using emerging transgenic “EV reporter” mouse models^10^ and careful study and manipulation of sEV populations in vivo. If CD81^+^ sEVs are indeed therapeutic, industrial-scale manufacturing of sEVs is already being utilized for numerous clinical trials, so CD81-enriched sEVs could be developed from “bench to bedside” rapidly to improve human metabolic health.
